# Gasdermin E mediates pyroptosis in the progression of hepatocellular carcinoma: a double-edged sword

**DOI:** 10.1093/gastro/goae102

**Published:** 2024-11-06

**Authors:** Yan Lu, Junnv Xu, Haifeng Lin, Mingyue Zhu, Mengsen Li

**Affiliations:** Hainan Provincial Key Laboratory of Carcinogenesis and Intervention, Hainan Medical University, Haikou, Hainan, P. R. China; Hainan Provincial Key Laboratory of Carcinogenesis and Intervention, Hainan Medical University, Haikou, Hainan, P. R. China; Department of Medical Oncology, Second Affiliated Hospital, Hainan Medical University, Haikou, Hainan, P. R. China; Department of Medical Oncology, Second Affiliated Hospital, Hainan Medical University, Haikou, Hainan, P. R. China; Hainan Provincial Key Laboratory of Carcinogenesis and Intervention, Hainan Medical University, Haikou, Hainan, P. R. China; Hainan Provincial Key Laboratory of Carcinogenesis and Intervention, Hainan Medical University, Haikou, Hainan, P. R. China; Department of Medical Oncology, Second Affiliated Hospital, Hainan Medical University, Haikou, Hainan, P. R. China; Institution of Tumor, Hainan Medical University, Haikou, Hainan, P. R. China

**Keywords:** hepatocellular carcinoma, *GSDME*, pyroptosis

## Abstract

Hepatocellular carcinoma (HCC) is the most common type of primary liver cancer worldwide. It usually develops due to viral hepatitis or liver cirrhosis. The molecular mechanisms involved in HCC pathogenesis are complex and incompletely understood. Gasdermin E (*GSDME*) is a tumor suppressor gene and is inhibited in most cancers. Recent studies have reported that, unlike those in most tumors, GSDME is highly expressed in liver cancer, and GSDME expression in HCC is negatively associated with prognosis, suggesting that GSDME may promote HCC. However, antitumor drugs can induce pyroptosis through GSDME, killing HCC cells. Therefore, GSDME may both inhibit and promote HCC development. Because functional studies of GSDME in HCC are limited, the precise molecular mechanisms of GSDME in liver cancer remain unclear. In this article, we have reviewed the role, related mechanisms, and clinical importance of GSDME at the onset and development of HCC to provide a theoretical foundation to improve the clinical diagnosis and treatment of liver cancer.

## Introduction

Hepatocellular carcinoma (HCC) accounts for 85%–90% of primary liver cancers and is one of the five most common malignant tumors worldwide [1–4]. Each year, >800,000 new cases and deaths related to HCC occur, making it the third-most common cause of cancer-related deaths worldwide [4, 5]. Due to the insidious onset of liver cancer, most diagnosed cases are in the middle to late stages of the disease, which greatly reduces treatment efficacy. Currently, primary treatments of HCC include surgery, chemotherapy, radiotherapy, and immunotherapy. However, due to its high incidence, recurrence, and metastasis, HCC still has suboptimal outcomes [6, 7].

**Figure 1.**
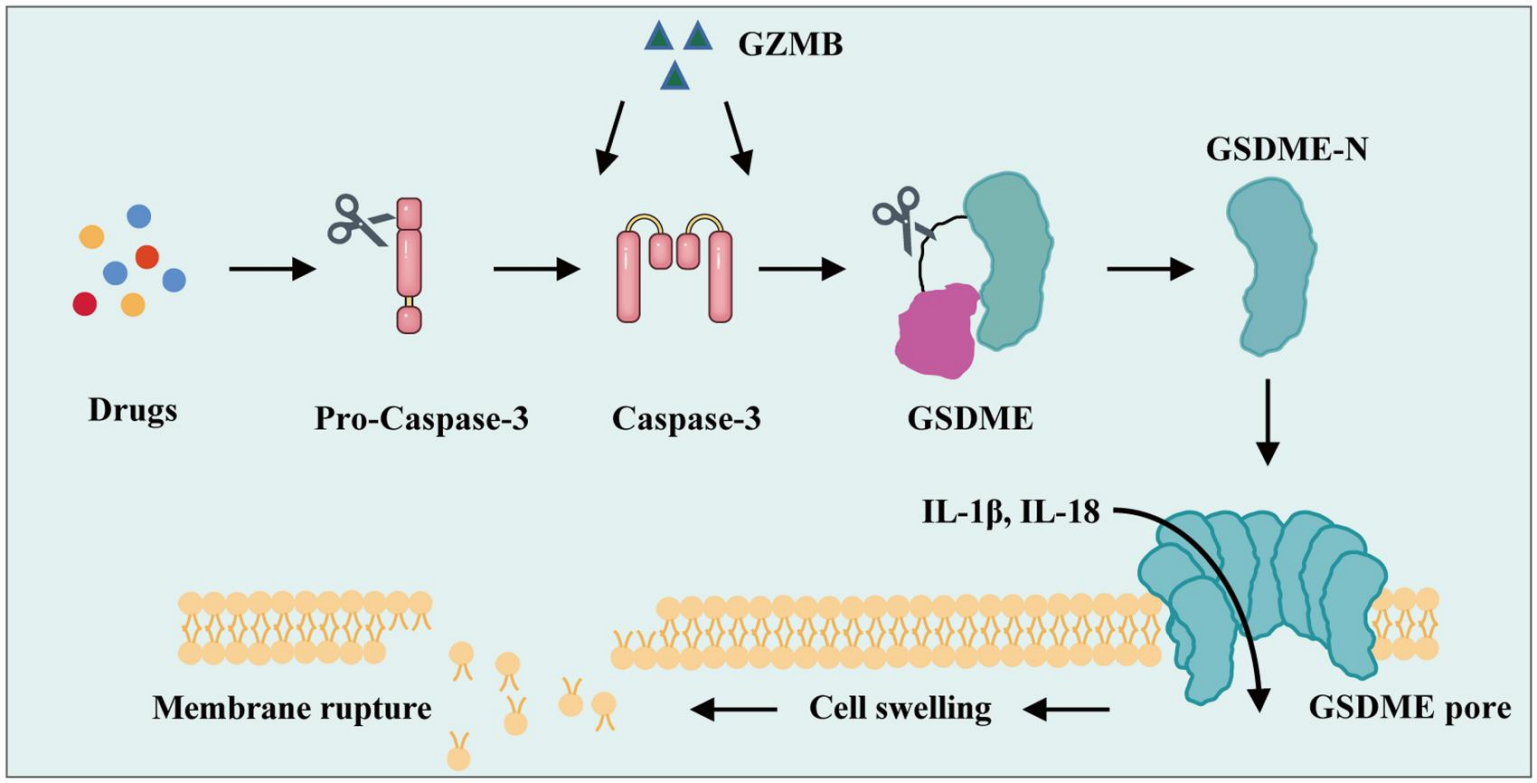
The molecular mechanism of Gasdermin E (GSDME)-mediated pyroptosis. Pyroptosis, orchestrated by GSDME, is triggered by drugs or granzyme B (GZMB). Caspase-3 that is activated by drugs and GZMB can cleave the GSDME protein, generating Gasdermin E N fragment (GSDME-N). The assembly of a pore complex in the plasma membrane by GSDME-N enables the efflux of interleukin-1β (IL-1β) and interleukin-18 (IL-18), culminating in osmotic imbalances, membrane expansion, and cellular distention and rupture.

Recent studies have shown that Gasdermin E (GSDME) and GSDME-mediated pyroptosis are critical in the occurrence and development of HCC [8–12]. Typically, GSDME is inactive in the cytoplasm and does not contribute to cell death. However, following cellular stress or damage, activated caspase-3 cleaves and activates GSDME, which releases its N- and C-terminal. The N-terminal binds to lipids on the cellular membrane and oligomerizes to form Gasdermin (GSDM) pores, which destroys the integrity of the cellular membrane, triggers the release of pro-inflammatory cytokines, such as interleukin 1beta (IL-1β) and interleukin 18 (IL-18), causing pyroptosis, and promotes the death of tumor cells [12, 13] (Figure 1). Extensive research has shown that pyroptosis can inhibit cancer occurrence and progression [9]. However, one study on colitis-associated colorectal cancer found that Gsdme^–/–^ mice experienced less weight loss and fewer instances of rectal prolapse, alongside smaller and fewer cancers [14]. Furthermore, GSDME-mediated pyroptosis can promote the occurrence and progression of colitis-associated colorectal cancer by releasing damage-associated molecular pattern molecules and high mobility group box 1 (HMGB1) [9, 14]. Moreover, multiple studies have shown that the role of pyroptosis in cancer depends on the type of pyroptosis and the specific mechanisms that are involved. Current research on GSDME-mediated pyroptosis in HCC is sparse; the relevant mechanisms of GSDME in HCC still need clarification. In light of this, we reviewed the current research on the expression and function of GSDME in HCC. We have provided a foundation for GSDME-related studies on HCC and suggested new strategies for diagnosing and treating liver cancer.

## GSDME structure and mediation of pyroptosis

*GSDME* was first identified in 1998 and initially named as deafness autosomal dominant 5 (*DFNA5*)—a gene associated with hereditary hearing loss. It is located on the short arm of chromosome 7 in humans and spans 496 amino acids [15]. The GSDME protein contains two distinct domains: a C-terminal peptide domain and an N-terminal Gasdermin domain. These domains perform various biological functions during GSDME activation. GSDME has a highly conserved amino acid sequence with high homology across species [16–18]. For example, the amino acid sequence similarity of GSDME proteins between humans and mice can exceed 88% [19]. Zebrafish have two forms of GSDME, each of which shares ∼50% sequence similarity with human GSDME [20].

GSDME-mediated pyroptosis is an inflammatory form of programmed cell death primarily triggered by caspases activation that cleaves the GSDME protein. The activation of the caspases process results in the N-terminal fragment of the Gasdermin protein undergoing oligomerization to form pores in the cellular membrane, leading to osmotic changes, membrane ballooning, and ultimately cell swelling and lysis [14, 19, 20] (Figure 1). Pyroptosis occurs much faster than apoptosis, releasing considerable amounts of inflammatory factors upon cell rupture. In April 2019, it was reported that the N-terminal pore-forming section of caspase-cleaved and activated GSDME punctured the mitochondrial membrane, promoting cytochrome C release and activating apoptosome, which enhanced mitochondrial apoptosis, promoted cancer cell death, and inhibited melanoma growth [21]. In March 2020, the GSDME-mediated pyroptosis pathway was found to inhibit the proliferation of gastric cancer, melanoma, colon cancer, and breast cancer cells by activating antitumor immunity [22]. In that study, the authors also found that granzyme B (GZMB) could directly cleave GSDME at D270, inducing pyroptosis independently of caspase-3. GZMB also indirectly induced GSDME-dependent pyroptosis by activating caspase-3 [22] (Figure 1). These results suggest that GSDME-mediated pyroptosis exerts antitumor effects. However, several studies have reported that GSDME-mediated pyroptosis paradoxically promotes the progression of colitis-associated colorectal cancer. Therefore, the role of GSDME-mediated pyroptosis may be cancer-type-specific.

## The expression of GSDME in HCC

Since GSDME-mediated pyroptosis was identified, studies have focused on its role in cancer. In most cancer tissues, including breast, colorectal, gastric, and bladder tissues, GSDME is expressed at lower levels than in normal tissues [23–25]. For example, Akino *et al.* [23] examined 89 gastric cancer tissue samples and found that ∼52% had low GSDME expression due to abnormal methylation of the *GSDME* promoter. Kim *et al.* [24] observed upregulated GSDME expression in HCT116, HT29, and DLD-1 colorectal cancer cell lines after treatment with the methylation inhibitor 5-azacytidine. They also determined that *GSDME* promoter hypermethylation was more common in colorectal tumor tissues than in normal colorectal tissues (65% vs 3%) [24]. The same methylation shift has also been reported in breast cancer [25]. In addition, transfection of exogenous *GSDME* into cancer cell lines with no or low GSDME expression reduced the proliferation and survival of cancer cells, whereas silencing of the *GSDME* gene significantly enhanced the cloning and invasion capabilities of cancer cells [24]. Therefore, it is believed that, in most cancer cells, *GSDME* is epigenetically silenced by excessive promoter hypermethylation. Thus, GSDME is considered a primary tumor suppressor.

On the other hand, De Schutter *et al.* [26] found that 10% of cancer cells exhibit elevated GSDME expression and its expression is higher in high-risk groups than in low-risk groups, suggesting that GSDME might promote specific types of cancer. For example, researchers used immunohistochemistry to analyse 105 esophageal cancer samples and 75 normal esophageal samples, and found that GSDME was higher in the cancer samples than in the normal esophageal samples [27]. In colitis-associated colorectal cancer, the GSDME protein levels were significantly higher than those in healthy controls [14]. HCC exhibits this pattern of GSDME expression. In 2021, Hu *et al.* [28] analysed the expression of GSDME in HCC tissues by using bioinformatics databases and reported a significant increase in GSDME mRNA levels in HCC tissues (Table 1). Subsequently, by using The Cancer Genome Atlas (TCGA) and Gene Expression Omnibus (GEO) databases, GSDME expression was found to be significantly upregulated in cancer tissues from HCC patients [29–32] (Table 1). Immunohistochemical analysis of liver cancer tissues also showed a significant increase in GSDME protein levels in cancer tissues compared with normal tissues [28–31] (Table 1). Western blotting analysis of GSDME expression in five liver cancer cell lines (HepG2, HuH7, LM3, SMMC-7721, and BEL-7402) revealed that GSDME is broadly expressed in HCC [33]. GSDME was also highly expressed in HepG2, HuH7, and MHCC97H liver cancer cells when using the same method [34] (Table 1). These results indicate that, unlike that in most cancers, the expression of GSDME in HCC is much higher than that in normal tissues.

**Table 1.** The expression of GSDME in HCC

Reference	Analysis	Technique	Expression	Prognosis
Gao X [35]	Gene expression	TCGA and GEO databases	In tumor tissue, GSDME was discovered to be upregulated	High expression of GSDME was associated with bad OS
Lai M [31]	Gene expression	TCGA and ICGC databases	GSDME was overexpressed in HCC tissues	GSDME exhibited a significant connection with a bad prognosis for patients
Shen Q [29]	Gene expression	TCGA database	GSDME was upregulated in tumor tissues	GSDME was associated with poor dismal survival
Zheng S [32]	Gene expression	TCGA and GEO databases	GSDME may play a tumor-promoting role in HCC	Low-expression group had better overall survival
Wang H [30]	Gene expression	TCGA database and validated using the ICGC and GEO databases	GSDME was increased in HCC tissues compared with normal tissues	In the GSDME high-expression group, HCC patients showed decreased survival rates
Fu XW [36]	Gene expression	TCGA and ICGC databases	GSDME was significantly upregulated	High expression of GSDME correlated with the poor survival of the HCC patients
	Protein expression	THPA database	The HCC tissues had higher protein levels of GSDME	
Hu K [28]	Gene expression	UALCAN database	GSDME was apparently increased in HCC tissue	Higher GSDME expression was associated with shorter OS and DSS in patients with HCC
	Protein expression	THPA IHC (tissue microarray)	GSDME was higher in HCC tissue compared with normal tissue	
Li G [37]	Gene expression	TCGA and GEO databases	The expression of GSDME in tumor tissues was higher than that in normal tissues	The prognosis for patients in the high-expression group was poor
	Protein expression	THPA	Higher GSDME expression in cancer	
Zhang X [33]	Protein expression	Western blot	GSDME was commonly expressed in HCC cell lines (L02, HepG2, HuH7, LM3, SMMC-7721, and BEL-7402)	
Shangguan F [34]	Protein expression	Western blot	GSDME was commonly expressed in HCC cell lines (HepG2, HuH7, HCCLM3, and MHCC97H)	

Normal samples were obtained from people without cancer.

HCC = hepatocellular carcinoma, GSDME = Gasdermin E, IHC = immunohistochemistry, TCGA = The Cancer Genome Atlas, GEO = Gene Expression Omnibus, THPA = The Human Protein Atlas, ICGC = International Cancer Genome Consortium, UALCAN = University of ALabama at Birmingham CANcer, OS = overall survival, DSS = disease-specific survival.

## GSDME affects the occurrence and development of HCC

### GSDME is associated with HCC staging and poor prognosis

*GSDME* is considered a tumor suppressor gene in most cancers due to its low expression [23–25]. In these tumors, individuals in the high-expression group exhibited a significantly higher 5-year survival rate than those in the low-expression group [24, 25]. However, some studies have reported that excessive GSDME expression is associated with low survival rates in head and neck squamous cell carcinoma, lung squamous cell carcinoma, and cholangiocarcinoma [38, 39]. In HCC, studies that utilized samples from a TCGA database reported a lower overall survival rate for primary liver cancer patients in the high-expression group than that in the low-expression group [35, 37], suggesting that GSDME is associated with a poor prognosis in HCC (Table 1). Similarly, GSDME is overexpressed in liver cancer tissues, and its expression is positively associated with tumor stage and negatively associated with patient survival [28, 29, 35] (Table 1). Based on these results, researchers speculate that *GSDME* is an oncogene that is associated with the staging and poor prognosis of liver cancer. However, the specific regulatory mechanisms involved in this process remain unclear.

### GSDME increases HCC sensitivity to antitumor drugs

Most antitumor drugs inhibit tumor progression by promoting apoptosis. However, tumor cells typically exhibit anti-apoptotic characteristics that promote drug resistance. Therefore, the use of non-apoptotic programmed cell death is critical in cancer therapy. Pyroptosis, which is a recently discovered form of cell death, has unique characteristics compared with apoptosis and may offer a new approach to minimizing chemotherapeutic resistance [40–42]. Cellular expression of GSDME determines whether cell death occurs through pyroptosis or apoptosis [43–45]. In high-GSDME-expressing tumor lines, the anticancer drugs cisplatin, 5-fluorouracil, and docetaxel activate caspase-3, which preferentially cleaves GSDME and induces pyroptosis [20, 46]. However, in some cancer cells, the promoter region of *GSDME* is in a state of low expression owing to abnormal hypermethylation, and caspase-3 activated by anticancer drugs cleaves the downstream apoptotic protein poly (ADP-ribose) polymerase (PARP) instead of GSDME, thereby initiating apoptosis. When GSDME-low-expressing cancer cell lines are treated with DNA methyltransferase inhibitors, GSDME expression is enhanced and the sensitivity of the cancer cells to drugs is correspondingly increased [24, 47]. These observations indicate that combining methyltransferase inhibitors with anticancer drugs can kill these types of cancer cells more effectively.

**Figure 2.**
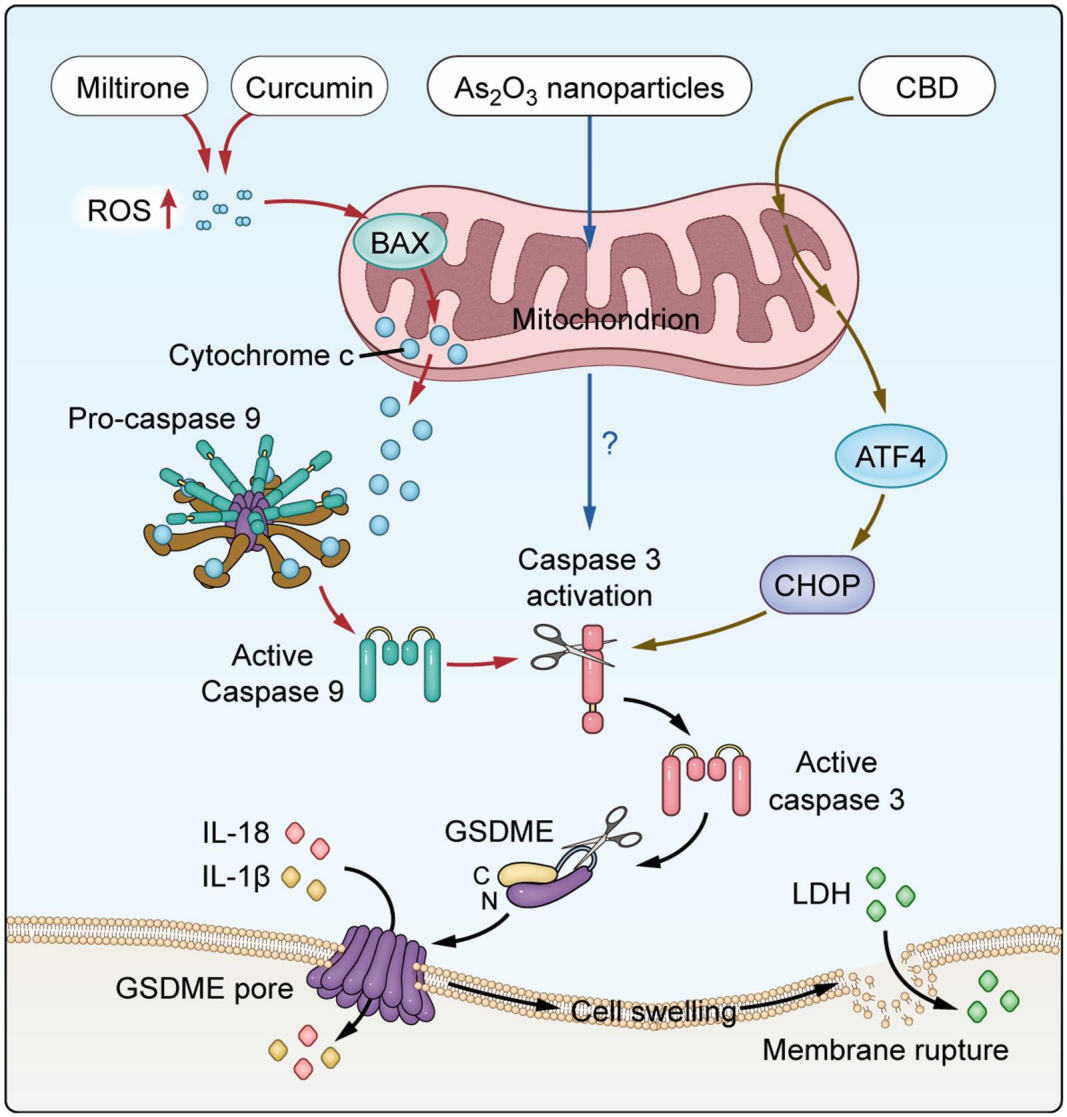
The molecular mechanism of drugs that promote hepatocellular carcinoma (HCC) cell death through Gasdermin E (GSDME)-mediated pyroptosis. Miltirone and curcumin, upon entering the cell, boost reactive oxygen species (ROS) levels, activating Bax, prompting cytochrome C release, and triggering caspase-9. This activates caspase-3, which cleaves GSDME to form membrane pores, inducing pyroptosis. Additionally, inflammatory factors including interleukin 18 (IL-18), interleukin 1β (IL-1β), and lactate dehydrogenase (LDH) are released. Arsenic trioxide nanoparticles (As_2_O_3_-NPs) act on the mitochondria, leading to the activation of caspase-3. Activated caspase-3 facilitates the cleavage of GSDME, which results in the removal of its inhibitory C-terminal domain and triggers pyroptosis. Cannabidiol (CBD) induces cellular and mitochondrial stress, leading to the activation of activating transcription factor 4 (ATF4) and subsequent upregulation of its target gene C/EBP-homologous protein (CHOP). This activates caspase-3, which cleaves GSDME, initiating pyroptosis.

Research has revealed that natural products such as miltirone and curcumin can induce the characteristics of pyroptosis, including cell swelling, lysis, lactate dehydrogenase (LDH) release, and PI-positive staining in HCC (Table 2). Miltirone causes increased cellular reactive oxygen species (ROS) production, activating Bax in mitochondria, which promotes cytochrome C release and the formation of the apoptotic complex via caspase-9 activation. Activated caspase-3 cleaves the GSDME protein, thereby initiating the pyroptosis pathway and inducing pyroptosis in liver cancer cells [33, 48, 49] (Figure 2). Studies have also reported a positive correlation between GSDME expression and the sensitivity of HCC cells to the first-line tyrosine kinase inhibitor lenvatinib. Further analysis indicated that lenvatinib upregulated the levels of GSDME in HCC cells and activated the N-terminal protein levels of GSDME, thereby inducing pyroptosis and promoting the death of liver cancer cells [36] (Table 2). Cannabidiol (CBD) has been suggested for the treatment of HCC by inhibiting aerobic glycolysis, which induces mitochondrial stress by regulating the ATF4–IGFBP1–Akt axis, to activate caspase-3, initiating the GSDME-mediated pyroptosis pathway [34] (Figure 2). Arsenic trioxide nanoparticles (As_2_O_3_-NPs) also can trigger GSDME-mediated pyroptosis in liver cancer cells expressing GSDME. In this process, activated caspase-3 initiates pyroptosis by cleaving the GSDME protein [50] (Figure 2). These findings suggest that GSDME-mediated pyroptosis can increase the sensitivity of HCC cells to antitumor drugs and promote cancer cell death. Therefore, GSDME plays a crucial role in the drug treatment process for liver cancer.

**Table 2.** The potential drugs for inhibiting HCC through GSDME-mediated pyroptosis

Drug	Molecular mechanism	Cell/animal model	Reference
As_2_O_3_ nanoparticles	Significantly upregulate the expression of GSDME-N	Xenograft tumor model with HuH7 cells, HuH7 and HepG2 cells	[50]
Miltirone	Elicits GSDME-dependent pyroptosis	HepG2 and Hepa1-6 cells	[33]
Curcumin	Induces GSDME-N-dependent pyroptosis	HepG2 cells	[48]
Lenvatinib	Upregulates the expression of GSDME and GSDME-N	HuH7 cells	[36]
Germacrone	Induces GSDME-dependent pyroptosis	HepG2 cells, Xenograft tumor model with HepG2 cells	[49]
Cannabidiol	Induces GSDME-N-dependent pyroptosis	HepG2 and MHHC97H	[34]

HCC = hepatocellular carcinoma, GSDME = Gasdermin E.

### GSDME influence on the HCC microenvironment

The GSDME protein plays a central role in modulating the tumor microenvironment. Upregulation of GSDME expression increases the number of natural killer and CD8^+^ T lymphocytes in the tumor microenvironment and enhances the phagocytic activity of tumor-associated macrophages against tumor cells. When GSDME is activated in tumors, it can transform “cold” tumors that are not recognized by the immune system into “hot” tumors that can be regulated by T cells and tumor-killing cells in the immune system; tumor growth is inhibited and cancer patients benefit from immunotherapy [22].

Single-cell sequencing of the HCC tumor microenvironment has demonstrated that GSDME expression is significantly increased in liver cancer tissues. GSDME is expressed in liver cancer cells and highly expressed in some immune cells. To further investigate the correlation between GSDME and immune cell infiltration, the Tumor Immune Estimation Resource database (version 2.0) was used to analyse the relationship between GSDME and immune cell infiltration. A positive correlation was found between GSDME and the infiltration of CD8^+^ T cells, B cells, neutrophils, and dendritic cells [29]. This indicated that GSDME might promote immune cell infiltration into HCC cells.

**Figure 3.**
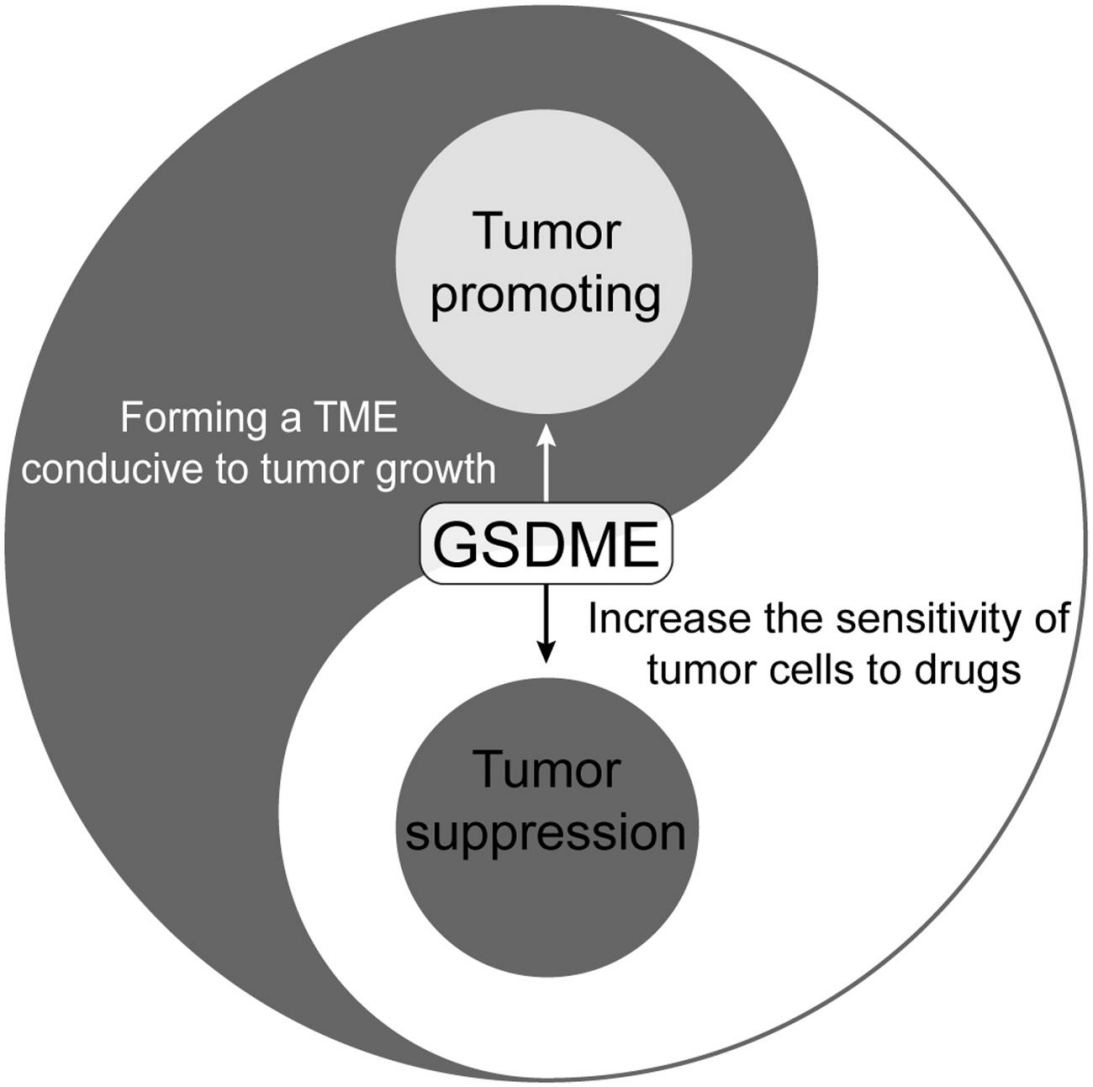
The double-edged-sword effect of Gasdermin E (GSDME) in hepatocellular carcinoma (HCC). GSDME-mediated pyroptosis enhances the sensitivity of HCC cells to drugs, resulting in cellular demise. However, this process also triggers the release of inflammatory cytokines that have the potential to foster a tumorigenic microenvironment within chronically inflamed tumors, thereby potentially facilitating disease progression.

Furthermore, several studies have found that GSDME-induced pyroptosis can lead to the release of large amounts of IL-1β and other inflammatory factors that can cause secondary damage to normal tissues. In some chronic inflammatory tumors, the long-term release of inflammatory factors forms a microenvironment that is suitable for cancer cell growth, which may induce normal cells around the tumor to become tumor cells and promote tumor progression [9, 36, 51]. Thus, GSDME-mediated pyroptosis may promote a chronic inflammatory microenvironment in HCC. Therefore, GSDME exhibits a double-edged-sword role in HCC. When GSDME mediates pyroptosis, it can increase the sensitivity of cancer cells to antitumor drugs, thereby promoting the death of tumor cells. On the other hand, the release of inflammatory factors during pyroptosis forms a microenvironment that is suitable for tumor growth in chronic inflammatory tumors, thereby promoting tumor progression (Figure 3). However, the coordinated mechanisms that balance these two processes remain unclear.

## Potential roles of GSDME in HCC diagnosis and treatment

### GSDME in HCC detection

In most tumor types, *GSDME* is typically identified as a pan-cancer biomarker due to abnormal DNA methylation [52–55]. Quantification and analysis of methylation site differences and methylation levels can be used to differentiate between different tumor types. Accurate prediction of cancer vs normal tissue across various tumor types was achieved with an area under the curve (AUC) of 0.87 by using combinations of six *GSDME* gene promoter CpGs [26]. Even when using only three CpGs, predictions in individual datasets resulted in AUCs that ranged between 0.80 and 0.95, demonstrating the substantial potential of *GSDME* as a biomarker for pan-cancer detection [26]. In a joint data analysis of >5,000 tumors and >700 control tissues, ∼75,000 combinations of CpG sites in the *GSDME* gene promoter were tested to determine whether they could be used to differentiate between different tissue types based on methylation. The results revealed maximal AUC values that ranged between 0.79 and 0.98 for predicting individual cancer types against all others, with prostate, thyroid, and colorectal cancers scoring the highest and esophageal cancer scoring the lowest. The highest AUC value for HCC was 0.89 [54]. Therefore, *GSDME* methylation has been proposed to be a reliable and specific clinical biomarker to determine liver cancer types. In addition, the DNA methylation levels of the *GSDME* gene in HCC tissues were studied by using the Human Disease Methylation Database and compared with those in normal liver tissues. A significant decrease in the methylation levels of *GSDME* was identified in HCC tissues, which is believed to be responsible for the increased GSDME mRNA expression [28]. These findings indicate that GSDME is promising for the early detection and diagnosis of HCC.

### GSDME in HCC treatment

Therapy with antitumor drugs is a critical treatment option for patients with advanced HCC. Antitumor drugs can promote HCC cell death by activating the GSDME-mediated pyroptosis pathway, which enhances their therapeutic effects. Because of the widespread GSDME expression in HCC cells and the higher expression of GSDME in liver cancer tissues than in normal liver tissues [27–30], some antitumor drugs, such as lenvatinib and miltirone, can activate caspase-3 and cleave GSDME, thereby initiating the pyroptosis pathway and promoting cancer cell death [32]. Therefore, liver cancer cells with high GSDME expression are more sensitive to pyroptosis that is induced by drugs. Therefore, effective activation of the GSDME-mediated pyroptosis pathway can increase sensitivity to antitumor drugs and reduce drug resistance in cancer cells. The promotion of liver cancer cell death via GSDME-mediated pyroptosis may offer a new strategy for HCC treatment.

## Conclusions and prospects

In conclusion, GSDME plays a crucial role in regulating the biological functions of HCC cells. Because of its high expression in tumor tissues, HCC is more sensitive to pyroptosis than normal tissues. Therefore, the induction of pyroptosis may have greater clinical value in HCC than in other tumor types. Pyroptosis is a double-edged sword that is associated with the occurrence and progression of HCC [9, 19, 28]. When antitumor drugs act on liver cancer cells and successfully activate pyroptosis mediated by GSDME, these tumor cells receive a lethal insult, effectively promoting tumor cell death. This indicates that GSDME has a potential cancer inhibitory effect and provides a new target for the treatment of liver cancer.

However, when cell pyroptosis occurs, cytokines such as IL-1β and interleukin 6 (IL-6) are released into the tumor microenvironment [12]. These cytokines regulate the transcription of inflammatory genes in the tumor microenvironment and affect immune cells in that microenvironment. Specifically, these cytokines activate a series of signaling pathways by binding to receptors on tumor and immune cells, regulating the inflammatory response in the tumor microenvironment. This inflammatory response can promote tumor growth and progression to some extent [56, 57]. Thus, GSDME has a double-edged-sword role in liver cancer, with potential anticancer effects and promoting tumor progression. Therefore, its dual role must be considered when using GSDME to treat liver cancer.

In future research, researchers need to comprehensively analyse the activation mechanism and functional principles of GSDME in cells and explore the profound impact of cell pyroptosis that is mediated by GSDME on the tumor microenvironment. It is critical to understand how cytokines such as IL-1β and IL-6 regulate the transcription of inflammatory genes in the tumor microenvironment and how these cytokines interact with immune cells in that microenvironment. Additional research will help to reveal the specific mechanism of double-edged-sword role of GSDME in liver cancer and provide new ideas and strategies for the treatment of liver cancer in the future.

Nevertheless, numerous scientific questions remain unanswered. Why is GSDME highly expressed in some tumors, such as HCC and colitis-associated colorectal cancer? GSDME is associated with HCC staging and poor prognosis; however, specific regulatory mechanisms are still unclear. In-depth exploration of the role of GSDME in the onset, development, and metastasis of HCC is urgently required. Given the double-edged-sword role of GSDME in HCC, its exploitation in clinical practice must be based on a careful analysis of its effectiveness and safety in treating HCC patients. Research on the role of GSDME in HCC progression may give rise to GSDME as a novel specific target for liver cancer detection, treatment, and prognosis, bringing new hope for HCC prevention and treatment.

## Authors’ Contributions

Y.L., J.X., and H.L. prepared the manuscript; M.Z. and M.L. designed the concepts and revised the manuscript. All the authors have read and approved the final manuscript.
